# Development of a trigger tool to identify harmful incidents, no harm incidents, and near misses in prehospital emergency care

**DOI:** 10.1186/s13049-024-01209-x

**Published:** 2024-04-29

**Authors:** Niclas Packendorff, Carl Magnusson, Kristoffer Wibring, Christer Axelsson, Magnus Andersson Hagiwara

**Affiliations:** 1 Prehospen–Centre for Prehospital Research, Faculty of Caring Science, Work Life and Social Welfare, University of Borås, Borås, Sweden; 2 Department of Prehospital Emergency Care, Sahlgrenska University Hospital, Gothenburg, Sweden; 3 Department of Ambulance and Prehospital Care, Region Halland, Sweden; 4 Institute of Health and Care Sciences, Sahlgrenska Academy, University of Gothenburg, Gothenburg, Sweden

## Abstract

**Background:**

Emergency Medical Services (EMS) are a unique setting because care for the chief complaint is given across all ages in a complex and high-risk environment that may pose a threat to patient safety. Traditionally, a reporting system is commonly used to raise awareness of adverse events (AEs); however, it could fail to detect an AE. Several methods are needed to evaluate patient safety in EMS. In this light, this study was conducted to (1) develop a national ambulance trigger tool (ATT) with a guide containing descriptions of triggers, examples of use, and categorization of near misses (NMs), no harm incidents (NHIs), and harmful incidents (HIs) and (2) use the ATT on randomly selected ambulance records.

**Methods:**

The ambulance trigger tool was developed in a stepwise manner through (1) a literature review; (2) three sessions of structured group discussions with an expert panel having knowledge of emergency medical service, patient safety, and development of trigger tools; (3) a retrospective record review of 900 randomly selected journals with three review teams from different geographical locations; and (4) inter-rater reliability testing between reviewers.

**Results:**

From the literature review, 34 triggers were derived. After removing clinically irrelevant ones and combining others through three sessions of structured discussions, 19 remained. The most common triggers identified in the 900 randomly selected records were deviation from treatment guidelines (30.4%), the patient is non conveyed after EMS assessment (20.8%), and incomplete documentation (14.4%). The positive triggers were categorized as a near miss (40.9%), no harm (3.7%), and harmful incident (0.2%). Inter-rater reliability testing showed good agreement in both sessions.

**Conclusion:**

This study shows that a trigger tool together with a retrospective record review can be used as a method to measure the frequency of harmful incidents, no harm incidents, and near misses in the EMS, thus complementing the traditional reporting system to realize increased patient safety.

**Supplementary Information:**

The online version contains supplementary material available at 10.1186/s13049-024-01209-x.

## Key messages

*What is already known on this topic:* The EMS system is potentially a high-risk environment for harmful incidents, and a reporting system could fail to detect adverse events; thus, new methods are needed.

*What this study adds:* This study adds an ATT to the clinical practice to complement the reporting system for the detection of harmful incidents, no harm incidents, and near misses and thus realize increased patient safety in EMS.

*How this study might affect research, practice, or policy:* The detection of harmful incidents, no harm incidents, and near misses in EMS could serve as a foundation for improving patient safety.

**Supplementary Information:**

The online version contains supplementary material available at 10.1186/s13049-024-01209-x.

## Background

Over the recent decades, the Emergency Medical Services (EMS) system in Sweden has transitioned from a transport service to an emergency care service that provides advanced care and patient assessments at the scene, including triaging to the most optimal level of care [1]. EMS clinicians can manage common complaints of patients of all ages in a variety of settings. As they operate in high-risk environments that involve the occurrence of adverse events (AE) and threats to patient safety [2], methods are needed for evaluating patient safety.

Various methods have been developed to measure patient safety and AE frequency [3]. Reporting systems are one of the most common methods; however, they are effective only if medical staff are aware of the occurrence of an AE [4, 5]. In EMS, a patient has few caregivers, and thus, the risk of an AE being missed is higher [6]. A structured retrospective record review (RRR) in combination with a trigger tool (TT) could complement a reporting system to detect up to 10 times more AEs [7]. Global Trigger Tool (GTT) is an example of a widely used tool of this type [8].

The report ‘To Err Is Human: Building a Safer Health System’ highlighted the need for working toward increased patient safety [9]. It was partly based on Harvard Medical Practice studies [9–11]. It revealed that 44,000–98,000 annual deaths at American hospitals were attributable to medical errors mainly due to drug complications, wound infections, and technical complications.

Few studies have investigated the incidence of AEs in EMS, although studies have adopted various methods. One explored the endotracheal intubation procedure performed by paramedics as it can easily be evaluated when arriving at the hospital; it found a fail rate of 25% [12]. Another study interviewed 15 EMS providers regarding events they recognized as an NM or AE; consequently, 50 events were categorized into errors prone to clinical judgement (54%), skill performance (21%), medications (15%), choice of destination (5%), and other (5%) [13].

One study conducted in Sweden used a TT originally developed in America for Helicopter Emergency Medicine Services (HEMS). It revealed a frequency of 4.3 AEs per 100 EMS missions [14]. The most common AE categorizes were unclear documentation and deviation from guidelines caused by mistakes made by the EMS clinicians. It also found higher AE incidence in patients with life-threating conditions.

To the best of our knowledge, no TT has been adapted for road-based EMS in western Europe. In this light, the present study aims to [1] develop a national ambulance trigger tool (ATT) with a guide containing descriptions of triggers, examples of use and categorization of near misses (NMs), no harm incidents (NHIs), and harmful incidents (HIs) and [2] use the ATT on randomly selected ambulance records.

## Methods

### Design

**Fig. 1:**
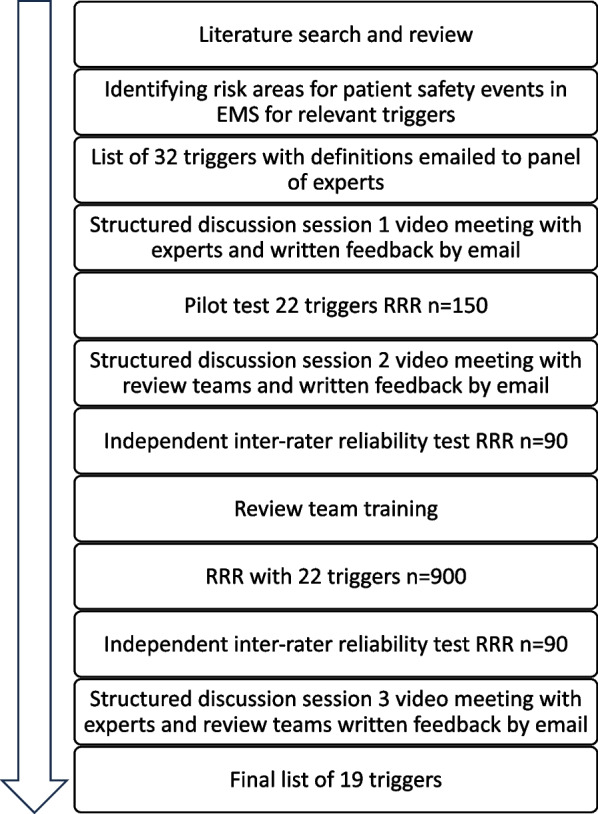
Flow chart of the development process of the EMS trigger tool. EMS, emergency medical services. RRR, retrospective record review

The ATT was developed by a stepwise approach including the [1] review of existing literature regarding patient safety and areas of risk for AEs in the prehospital field [2] expert panel discussions with adaptation of the ATT through a video link, and [3] clinical evaluation of the ATT through RRR (Fig. 1).

### Setting

The EMS system in Sweden is funded by taxes across regions, resulting in local variations in guidelines and documentation systems. At a national level, it was specified that in each ambulance, one of the two EMS clinicians must be a registered nurse (RN) often with a degree of master in ambulance/intensive/anaesthesiologic care.

We invited the majority of EMS organizations and three services were recruited across Sweden: an urban service with 85,000 EMS missions each year and a median mission time of 70 min and two urban–rural mixed services with 53,000 and 41,000 EMS missions each year and median mission times of 78 and 66 min, respectively. Two of these services participated in the first two sessions of structured discussions and the other one participated in the third.

### Terminology and definitions

We used the World Health Organization (WHO) terminology for incidents including NM, NHI, and HI [15, 16] and categorized the incidents according to the National Coordination Council for Medication Error Reporting and Prevention index (NCC MERP) [17].

An NM incident (e.g. lack of documentation) neither affects nor harms the patient but poses the risk of an error and is categorized as (A) or (B) according to the NCC MERP.

An NHI (e.g. omission of electrocardiogram (ECG) in patient with chest discomfort not diagnosed with acute coronary syndrome) affects the patient but does not cause harm. It is categorized as (C) ‘An incident that affected the patient but did not cause any harm’ or (D) ‘An incident that affected the patient and demanded observation or treatment to assure that no harm occurred’.

NMs and NHIs are traditionally not used when using TTs but could be valuable in terms of evaluating the clinical setting by receiving information about commonly occurring NMs and NHIs [18].

An HI (adverse event; e.g. omission of ECG in patient with chest discomfort and later diagnosed with ST-elevated myocardial infarction, thus delaying time to causal treatment) harms the patient. It is categorized as (E) ‘Contributed to or resulted in temporary harm and required intervention’; (F) ‘Contributed to or resulted in temporary harm requiring outpatient care, readmission, or prolonged hospital care’; (G) ‘Contributed to or caused permanent patient harm’; (H) ‘An event that required lifesaving intervention within 60 min’; or (I) ‘Contributed to the patient’s death’.

### Step 1. Literature review

Existing TTs for inpatients, homecare, paediatric care, and psychiatric care presented by the Swedish Association of Local Authorities and Regions (SKR) were reviewed for developing the new ATT [19]. To cover a prehospital context and to identify risk areas for patient safety, PubMed, Cinahl and Medline was searched with the following keywords: patient safety, prehospital, ambulance, trigger tool, and adverse event. Headlines and abstracts were read and included with the following criteria (1) a description of incidents regarding patient safety in EMS measured with various methods, (2) ≥ 18 years of age, (3) not published before 1980.

### Step 2. Expert panel and Structured discussions

Five experts, including RNs and medical doctors (MDs), were invited via email to contribute during the discussions. The RNs were eligible to participate in the development of the ATT if they had 10 years’ EMS clinical experience, previous experience in patient safety in an EMS organization, and experience regarding the usage of a TT. The MDs were recruited because of their knowledge and experience in the methodology of developing TTs. They were also responsible for patient safety in the healthcare system at a regional or national level within their organization. All members of the panel where confident in other methods used in the patient safety context such as incident reporting or root cause analysis. The experts were recruited on the authors opinions who was suitable but also depending on personal interest from the experts.

Three sessions of structured discussions was employed to reach a consensus among a panel of experts regarding the ATT, where the collective opinion of the group is deemed stronger than that of each individual [20]. An expert is known to be a specialist in their field, an individual who has knowledge on a specific matter [21, 22]. During the three sessions, the discussions was structured as follows: each definition and trigger were analysed using a shared screen view for language, clinical relevance, and user-friendliness. The document was seen by all experts, and the removal, adaptation, or combination of triggers was done instantly. Each correction was approved by the experts before moving on to the next trigger. The session was video recorded so that the session would be viewable again and the corrections were emailed to the experts with the ability to give written feedback. Consensus was considered achieved when all experts had approved the ATT via email, and no further feedback was provided by them in writing.

### Step 3. RRR process and Review teams

The measurement of patient safety with TTs requires access to patient records. In this light, review teams consisting of an RN and an MD were formed, where the RN starts with a primary review of the patient record with guidance of a list of triggers. A trigger can be a clue that an incident has occurred. Each trigger comes with a definition containing criteria that need to be fulfilled for the trigger to be considered positive.

The RN searches the records for positive triggers, reviews them for a potential incident, and classifies the incident according to the three first steps (AB, C, D) of the NCC MERP [17]. Records with an incident graded C and D undergo a secondary review by the MD, who assesses them for an HI. If an HI is found, the MD classifies it according to the last five steps (E, F, G, H, I) of the NCC MERP. The MD also classifies the type of HI and whether it is preventable by using the following scale: (1) ‘not preventable’; (2) ‘probably not preventable’; (3) ‘probably preventable’, and (4) ‘certainly preventable’.

The three EMS organizations each formed a review team consisting of a MD and a RN. The MD was in medical charge of their organization, and the RN had extensive experience of working in the EMS within this organization. One organization used two RNs having comparable experience in working in the EMS in the primary review.

In a pilot study before session two of the structured discussions, two review teams from two different organizations randomized 150 records and performed an RRR to gain experience in using the ATT.

Before session three of the structured discussions, three review teams from different organizations randomized 900 records and performed an RRR to further evaluate the ATT and to receive frequencies of positive triggers and incidents from their organizations. Before the RRR the review teams received training in how to extract and randomize data, how to use the ATT within Microsoft Access®, definitions of common terminology regarding a TT (e.g. positive trigger, NM, NHI, and HI), and examples with fictitious records. The reviewers had no previous experience in using a TT.

The criteria for inclusion in both RRR was age ≥ 18 years and a primary mission where a patient assessment takes place. Children were excluded in this study because several studies have shown that different triggers are required to study the paediatric population in EMS [23, 24]. There are plans to develop a set of triggers adapted for children.

### Analysis

The positive triggers, NMs, NHIs, and HIs were presented in a frequency-based manner according to both the WHO and the NCC MERP. Each member of the review teams graded the triggers according to clinical relevance, comprehensibility, and utility with a 4-point Likert scale, where 1 = not relevant, 2 = somewhat relevant, 3 = quite relevant, and 4 = highly relevant after the RRR. The item-level content validity index (I-CVI) was calculated for each trigger by summing the number of reviewers grading trigger 3 or 4 and divided by the total number of reviewers. I-CVI of 0.80 or higher was considered highly relevant [25]. The positive predictive value (PPV) was calculated for each trigger by how many times the trigger resulted in a near miss, no harmful or harmful incident divided by the total times the trigger was found multiplied by 100 [26]. The Mersenne Twister algorithm was used for randomizing the records [27].

Two sessions of independent inter-rater reliability (IRR) tests between two primary reviewers were conducted, with the triggers serving as the variable for testing. The total outcome of positive triggers was summed up and analysed in a confusion matrix with Cohen’s kappa (Table 3) [28]. Kappa values of 0.21–0.40, 0.41–0.60, 0.61–0.80, and 0.81–1.00 were respectively considered fair, moderate, substantial, and almost perfect [29]. The ATT produces an overrepresentation of negative triggers when no incidents have occurred and creates a prevalence problem which causes Cohen’s kappa to be low [28, 30]. Therefore, Cohen’s kappa was complemented with a prevalence-adjusted and bias-adjusted kappa (PABAK) [30]. The interpretation of PABAK and Cohen’s kappa is the same [31]. All analyses were performed with R studio version 2023.03.0 + 386.

## Results

### Literature review

The review of previous TTs from SKR and the literature search using PubMed, Cinahl and Medline revealed 32 triggers (refer to supplement 1 and 2). The structure of the ATT was constructed to be the same as that of previous TTs from SKR, and each of the 32 triggers was defined and received definition for the trigger to be positive.

### Structured discussion session one

The 32 triggers and definitions were emailed to the five experts two weeks before the planned session. After session one, a total of ten triggers were removed, reducing the number to 22. This was achieved by removing clinically irrelevant triggers or combining several triggers into one. These excluded triggers concerned areas that were either fully covered by other triggers after modifying their definition or were outside the scope of EMS. The majority of the removed triggers were related to EMS organizational quality aspects and were deemed beyond the scope of the EMS trigger review within the EMS record. Examples include triggers related to resource allocation, dispatch actions, or inactions (refer to supplement 2).

### RRR with 150 records

To receive feedback from the clinical professionals regarding the 22 triggers, they were evaluated with RRR in clinical practice. Two EMS organizations formed review teams consisting of an RN as a primary reviewer and an MD as a secondary reviewer. The review teams were asked to use the ATT when reviewing randomized records from their organization. The aim of the review teams was to evaluate the clinical relevance, utility, and comprehensibility of the ATT in a clinical setting but also the usability of the digital database. The teams also documented time in minutes for each record reviewed. The ATT presented the following top three positive triggers from the 150 records, B1 Deviations from treatment guidelines in 47 (31%), A1 Incomplete documentation in 33 (22%) and B6 The patient is non conveyed after EMS assessment in 34 (22.7%). The review time was median 5 min per record with a range from 2 to 18 min. These experiences and result showed that the method was feasible, and the digital database was sufficient which served as a foundation for the next session of discussion.

### Structured discussion session two

Session two consisted of members of the review teams from the RRR with 150 records. The review teams used their knowledge, experience, and result from the previously performed RRR to further adapt the definitions of the triggers. This round resulted in changes in trigger definitions; however, no trigger was removed or added for example A1 Incomplete documentation received five criteria to be considered positive and B5 Inconsistency between the EMS clinicians and emergency physicians assessment and triage received criteria to be positive if the patient were taken from the ED to definitive care (thrombolysis, percutaneous coronary intervention, intensive care) directly after the physicians assessment. (refer to supplement 2).

### IRR session one based on 90 records

The first session of IRR testing between two primary reviewers was carried out on 90 records (Table 1). Cohen’s kappa *k* = 0.5 and PAPAK *k* = 0.89.

**Table 1:** IRR^a^ between two primary reviewers’ triggers when reviewing identical patient records (*n* = 90)

Session one		RN^b^ 1		Session two		RN^b^ 1	
		Positive	Negative			Positive	Negative
RN^b^ 2	Positive	59	69	RN^b^ 2	Positive	31	53
	Negative	34	1818		Negative	60	1836
	Cohen’s D 0.5	Accuracy 0.95	PABAK^c^ 0.89		Cohen’s D 0.3	Accuracy 0.94	PABAK^c^ 0.88

^a^Inter-rater reliability

^b^Registered nurse

^c^Prevalence-adjusted and bias-adjusted kappa

### RRR with 900 records

The final use of the ATT consisted of three EMS organizations, where each organization formed review teams. The RNs categorized the records meeting the inclusion criteria by each month and randomized 25 per month in subject for review; 300 records per organization were used. Table 2 lists the demographics, and Table 3 lists the frequencies of positive triggers with PPV grouped by incidents, and Table 4 lists the classification of incidents according to the NCC MERP and WHO. Nine records were excluded from one mixed organization for not meeting the inclusion criteria. After the RRR, the members of the review teams received an online form consisting of the 4-point Likert scale in order to calculate I-CVI for each trigger presented in Table 5.

**Table 2:** Demographics of the retrospective record review (*n* = 891)

	Mixed organization 1 (*n* = 300)	Mixed organization 2 (*n* = 291)	Urban organization (*n* = 300)
**Age Median (quantile)** ^**a**^	73 (52–81)	69 (49–81)	63 (41–79)
**Sex ** ***n *** **(%)**			
Female	152 (50.7)	154 (52.9)	155 (51.7)
Male	148 (49.3)	137 (47.1)	145 (48.3)
**Dispatcher priority ** ***n *** **(%)**			
Prio 1^b^	138 (46)	106 (36.4)^a^	118 (39.3)
Prio 2	150 (50)	169 (58.1)	166 (55.3)
Prio 3	12 (4)	15 (5.2)	16 (5.3)
**Prehospital triage colour according to RETTS-A** ^**c**^ ***n*** **(%)**			
Red	23 (7.7)	35 (12) ^b^	26 (8.7) ^a^
Orange	115 (38.3)	91 (31.3)	67 (22.3)
Yellow	108 (36)	91 (31.3)	111 (37.0)
Green	54 (18)	51 (17.5)	54 (18)
Blue	0 (0)	3 (1.0)	2 (0.7)
**Initial assessment of level of care ** ***n *** **(%)**			
Hospital	251(84)	225 (77.4) ^c^	209 (69.7)
Stay on scene	49 (16)	65 (22.3)	91 (30.3)
**Mode of transport to hospital ** ***n *** **(%)**			
Ambulance	248 (83)	216 (74.2) ^c^	183 (61)
Patient transport	1 (0.003)	1 (0.3)	13 (4.3)
Seated transport	1 (0.003)	2 (0.7)	9 (3)
Single responder	0 (0.0)	0 (0.0)	3 (1)
Own transportation	1 (0.003)	6 (2.1)	1 (0.3)
**Top five prehospital field assessment according to RETTS-A** ^**c**^ ***n*** **(%)**			
Respiratory distress/dyspnoea/breathing difficulties	32 (10.7)	35 (12.0)	20 (6.7)
Chest thoracic pain	23 (7.7)	32 (11.0)	34 (11.3)
Abdominal/flank pain	17 (5.7)	24 (8.2)	22 (7.3)
Injury/head trauma	15 (5.0)	12 (4.1)	16 (5.3)
Infection	15 (5.0)	25 (8.6)	21 (7.0)
Dizziness	14 (4.7)	14 (4.8)	11 (3.7)
^a^ 25^th^ and 75^th^ quantiles ^b^ Prio 1 (lights and sirens) ^c^ Rapid Emergency Triage and Treatment System-Adult		^a^ 1 missing ^b^ 20 missing ^c^ 1 missing	^a^ 40 missing

**Table 3:** Positive triggers and Positive predictive value (PPV) for each trigger grouped by near miss, no harmful incident and harmful incident after reviewing patient records in the three EMS organizations (*n*=891)

	Positive triggers detected in primary review *n *(%)	Positive trigger related to near miss *n *	PPV near miss (%)	Positive trigger related to no harmful incident *n*	PPV no harmful incident (%)	Positive trigger related to harmful incident *n*	PPV harmful incident (%)
A1 Incomplete documentation	128 (14.4)	118	92,1	7	5,4	0	0
A2 Response time >20 min priority 1 (lights and sirens)	26 (2.9)	21	80,7	1	3,8	1	3,8
A3 Time on site >10 min in case of life-threatening conditions	19 (2.1)	16	84,2	0	0	0	0
A4 Weather and environment affect patient care	0 (0)	0	0	0	0	0	0
A5 Breakdown or faulty/missing equipment	6 (0.7)	6	100	0	0	0	0
A6 Shortage of ambulance resources	3 (0.3)	3	100	0	0	0	0
A7 Other	0 (0.0)	0	0	0	0	0	0
B1 Deviations from treatment guidelines	271 (30.4)	238	87,8	24	8,8	1	0,3
B1A Assessment/Interventions according to SX-ABCDE ^a^	96 (10.8)	84	87,5	11	11,4	1	1,0
B1B Assessment/Interventions in specific conditions	24 (2.7)	16	66,6	8	33,3	0	0
B1C Absence of measured vital signs	70 (7.9)	60	85,7	6	8,5	0	0
B1D Absence of relevant clinical examination	144 (16.2)	131	90,9	9	6,25	0	0
B2 Physical harm during patient transport	0 (0.0)	0	0	0	0	0	0
B3 Deterioration of Patients Condition during Transport	1 (0.1)	1	100	0	0	0	0
B4 Telephone interpreter has not been used in case of language deficiency	12 (1.3)	10	83,3	1	8,3	0	0
B5 Inconsistency between the EMS clinicians and emergency physicians assessment and triage	8 (0.9)	4	50	4	50	0	0
B6 The patient is non conveyed after EMS assessment	185 (20.8)	102	55,1	5	2,7	1	0,5
B6 Patient return within 72 h	28 (15.1)	19	67,8	4	14,2	1	3,5
B7 Alternative mode of transport to definitive care	32 (3.6)	13	40,6	0	0	0	0
B8 Ambulance destination deviates from local guidelines	2 (0.2)	1	50	1	50	0	0
L1 Unfavourable/Inappropriate drug treatment	2 (0.2)	0	0	2	100	0	0
L2 Mix-up of drugs	0 (0.0)	0	0	0	0	0	0
L3 Shortage of medicines due to absence/expiry date	0 (0.0)	0	0	0	0	0	0

^a^ Scene safety, eXanguinating bleeding, Airway, Breathing, Circulation, Disability, Exposure

**Table 4:** Classification of incidents after reviewing patient records in the three EMS ^a^ organizations (*n* = 891)

**Incidents according to NCC MERP** ^b^ ***n *** **(%)**		**Incidents according to WHO** ^c^ ***n *** **(%)**	
		No incident	492 (55.2)
AB	364 (40.9)	Near miss	364 (40.9)
C	29 (3.3)	No harm incident	33 (3.7)
D	4 (0.4)		
E	1 (0.1)	Harmful incident	2 (0.2)
F	0 (0.0)		
G	1 (0.1)		
H	0 (0.0)		
I	0 (0.0)		

^a^Emergency Medical Services

^b^National Coordination Council for Medication Error Reporting and Prevention index

^c^World Health Organization

**Table 5:** Triggers with I-CVI ^a^ after reviewing patient records in the three EMS ^b^ organizations (*n*=891)

	Clinical relevance (CVI)	Comprehensibility (CVI)	Utility (CVI)
A1 Incomplete documentation	1.00	1.00	1.00
A2 Response time >20 min priority 1 (lights and sirens)	0.66	1.00	0.83
A3 Time on site >10 min in case of life-threatening conditions	0.66	1	0.66
A4 Weather and environment affect patient care	0.33	0.83	0.33
A5 Breakdown or faulty/missing equipment	1	0.83	0.83
A6 Shortage of ambulance resources	0.66	0.66	0.5
A7 Other	0.83	0.5	0.5
B1 Deviations from treatment guidelines	1	1	1
B1A Assessment/Interventions according to SX-ABCDE ^c^	1	1	1
B1B Assessment/Interventions in specific conditions	1	1	1
B1C Absence of measured vital signs	0.83	1	0.83
B1D Absence of relevant clinical examination	1	0.83	1
B2 Physical harm during patient transport	0.66	1	0.83
B3 Deterioration of Patients Condition during Transport	0.66	0.83	0.83
B4 Telephone interpreter has not been used in case of language deficiency	0.66	0.83	0.66
B5 Inconsistency between the EMS clinicians and emergency physicians assessment and triage	1	0.83	1
B6 The patient is non conveyed after EMS assessment	1	0.83	0.83
B6 Patient return within 72 h			
B7 Alternative mode of transport to definitive care	1	1	1
B8 Ambulance destination deviates from local guidelines	1	1	1
L1 Unfavourable/Inappropriate drug treatment	1	1	1
L2 Mix-up of drugs	0.83	0.83	0.66
L3 Shortage of medicines due to absence/expiry date	0.66	0.83	0.5

^a^ Item-level Content Validity Index

^b^ Emergency Medical Services

^c^ Scene safety, eXanguinating bleeding, Airway, Breathing, Circulation, Disability, Exposure

### IRR session two based on 90 records

In the second session of IRR testing, the same primary reviewers as those in the previous session conducted an RRR of the same 90 records, producing Cohen’s kappa *k* = 0.3 and PAPAK *k* = 0.88 (Table 1).

### Structured discussion session three

In session three, both the experts and review teams (in total 12) were invited for a video meeting. Because of difficulties in gathering both the panel of experts and review teams, this round was divided into two groups with 10 participants. Two weeks before the session was scheduled, the experts and review team members received the frequencies from the previous RRR, including the I-CVI and IRR results. In this round, the number of triggers was reduced to 18 (refer to supplement 2), and the process of categorizing incidents was altered as follows. The primary examiner decided whether positive triggers in the patient records contributed to an incident, number of incidents, and whether the incident affected the patient with a risk of harm or not. Incidents that did not affect the patient or entail any risk of harm were classified according to the categories AB and C by the primary reviewer. An incident with a risk of the patient being harmed is secondary reviewed by a MD and no classification of the incident is made by the primary reviewer but left to the secondary reviewer. The secondary reviewer assessed incidents with a risk of harm and decided whether the patient was harmed or not. If the patient was not harmed, categories AB, C, and D were used. If the patient was harmed, the type of harm and degree of severity were assessed according to the categories E to I. Refer to supplement 3 for a translated manual of the final ATT.

## Discussion

Three structured discussion sessions were conducted with an expert panel, complemented by RRR, to develop the ATT. In this study, the sessions were held via video meetings, with trigger definitions displayed on a shared screen. The moderator made real-time changes to the document in consensus with the panel of experts. Several methods exist to achieve consensus among expert panels, with one of the most commonly used being the Delphi technique [32]. Anonymity is a key feature of conducting a Delphi survey, as it is argued to mitigate biases caused by hierarchy or individual dominance within the expert panel [33]. However, the technique has been modified in various ways, leading to questions regarding its methodology [34, 35].

The original methodology employs a paper-based approach, where questionnaires are distributed to a panel of experts [20]. The researcher analyzes the responses to generate statements and questions, which are then rated by the experts in subsequent rounds [32]. The original Delphi has been suggested to be ineffective and error-prone [36].

The methodology used in this study has been successfully employed in previous studies for developing TTs [26, 37]. We identify several advantages with this methodological approach compared to conducting a traditional Delphi survey. In Sweden, there is a limited number of experts regarding the use of trigger tools, especially within the context of EMS. While the size of a traditional Delphi panel can vary from 10 to 1000 [33], there is no standard number [38]. In this study, experts were selected based on personal invitations from the author using specific criteria (refer to the method section), which may impact the homogeneity of the group. A diverse group may lead to a broader discussion, whereas a homogeneous group may result in more reliable outcomes, depending on the study's aim [33]. However, including experts who are not knowledgeable about patient safety and the use of trigger tools in EMS could potentially negatively influence the identification of triggers by excluding those that may identify incidents. We believe that the panel size, complemented by literature review RRR in this study, was sufficient to develop a comprehensive TT covering most aspects of patient safety incidents in EMS.

The use of video meetings to conduct structured discussions was considered a strength as it allowed experts from various geographical locations to participate, thus enabling a higher degree of participation and reducing dropouts. While face-to-face discussions lacked anonymity, literature suggests that complete anonymity is challenging to achieve since the researcher knows the experts and there may be different relations between the experts not known by the researcher [34]. Due to the complexity of the triggers and definitions, we believe that there was an advantage in discussing the triggers face-to-face in a qualitative manner, rather than making them quantifiable using surveys. We utilized RRR as a quantitative component to gain clinical experience with the ATT to be used later in the structured discussions.

Face-to-face discussions are also utilized in other methodologies such as focus groups or the nominal group technique [39, 40]. The lack of anonymity also appears to motivate experts to participate in the sessions [41]. Experts and review teams participating in the structured discussions were given the opportunity to provide written feedback individually after the meeting, allowing for corrections if issues regarding lack of anonymity were present during the discussions. Our experience from the current discussions is that no member dominated the discussion, and all were given the opportunity to express their opinion. The written feedback provided less input to the TT compared to the structured discussions.

The use of a TT comes with reliability issues between the reviewers, and studies have shown that even experienced review teams will review records differently [42]. The IRR also seems to decrease if the triggers are subjective [43]. To increase the IRR, it is recommended to use team training, a two-way review process, and reviews in consensus instead of independently [44, 45]. This study used team training, a two-way review process, as well as a manual with definitions of the triggers to increase the IRR.

The most common triggers identified in the records were incomplete documentation, deviation from guidelines, and termination of patient care after the EMS clinician’s assessment. Incomplete documentation was found in 14% of patient records; although it might not contribute to an HI, it contributes substantially to NM, thus emerging as an important area for improvement in the EMS. One study [46] simulated the actions of EMS clinicians during medical or traumatic emergency care of a patient. The video-recorded actions were later compared with the documentation, and they revealed missing documentation in 22% of medical cases and 14% of traumatic cases. Incomplete documentation could be caused by several reasons; a study has shown that incomplete documentation poses a risk of errors when transferring a patient from the EMS to the emergency department (ED) because of differences in the verbal report and what is later recorded [47].

Deviation from guidelines was found in 30% of records and was further categorized as assessment/interventions according to SX-ABCDE (Scene safety, eXanguinating bleeding, Airway, Breathing, Circulation, Disability, Exposure), where (11%) of the deviations were found, along with absence of measured vital signs (8%), and absence of relevant clinical examination (16%). One record could have several positive triggers in the categorization of deviation from guidelines which affect patient care negatively. The assessment/interventions according to SX-ABCDE was positive if the EMS examiner failed to assess the scene safety or the patient’s XABCDE or failed to address issues according to the algorithm. The ABCDE approach is widely accepted by expert consensus in the medical, surgical, and anaesthetics field to improve the quality and speed of patient treatment [48].

The absence of measured vital signs was positive if the record failed to display the common vital signs, rate of breathing, saturation, blood pressure, pulse, and temperature. Blood-glucose was added if indicated for loss of consciousness or seizures. A study of non-conveyed patients showed vital signs data was missing in 6%–19% of patient records [49]. Studies have shown that both a lack of on-scene vital signs for trauma patients and a failure to notice deviations of vital signs at the ED were associated with increased mortality [50–52].

The absence of relevant clinical examination was positive if a patient did not receive appropriate examination in relation to the chief complaint. For example, if the patient’s chief complaint was chest pain and they were not examined with an ECG, or the chief complaint was abdominal pain but they did not receive an abdominal examination (e.g. auscultation, palpation). The prehospital ECG showed abnormalities in 19% of cases in comparison with the ECG at the ED, thus potentially affecting the prospective care of the patient [53, 54].

The trigger ‘patient care is terminated after the ambulance nurse’s assessment’ was found in 20.8% of cases, and the trigger ‘patient contacted the ambulance or ED within 72 h for the same symptoms’ was found in 15.1% of cases, which correlates with previous findings [1, 55].

The reviewers identified HI in two records (0.2%), NHI in 33 records (3.7%), and NM in 366 records (40.6%). A previous study [2] reported a frequency of 4.3% (46 out of 1080) in prehospital records using a different terminology with the AE potential for harm (43 out of 1080) and AE with harm identified (3 out of 1080). The different terminology used makes it difficult to make direct comparisons between the results of the studies; however, the HI in this study shows a similar result as AE with harm identified in the previous study.

### Strengths and limitations

To our knowledge, this is the first study of EMS in which positive triggers are evaluated by a primary and secondary reviewer according to the NCC MERP [17]. This enables the categorization of the triggers into NMs, NHIs, and HIs according to the WHO, which could serve as a foundation for improvement within a given EMS organization [18].

One limitation in this study could be the number of participants of 12 which could affect the results in not recognizing possible areas of risk for patient safety in the prehospital environment. One limitation in reviewing records from the own organization thus creates a risk for underestimating the occurrence of positive triggers, NHs, NHIs, and HIs. Another limitation could be the failure to include a rural organization with a longer time of transport to participate. None of Sweden’s rural organizations agreed to participate in this study.

## Conclusions

The EMS environment poses significant risks to patient safety, yet it remains inadequately studied. Conventional incident reporting systems often fall short in capturing these risks, necessitating a multifaceted approach to enhance patient safety. Our study introduces a tailored trigger tool for EMS, demonstrating its potential in identifying safety-threatening incidents. This tool provides a foundation for future research, offering a systematic means of incident detection and refinement. Beyond its immediate utility in incident detection within EMS, the trigger tool engenders a framework conducive to ongoing refinement and elucidation of trigger parameters and definitions, thus facilitating a deeper understanding of safety dynamics within the EMS.

### Supplementary Information

**Supplementary Material 1.**

**Supplementary Material 2.**

**Supplementary Material 3.**
