# Supplementary material for: Development of a trigger tool to identify harmful incidents, no harm incidents, and near misses in prehospital emergency care
Source: Scand J Trauma Resusc Emerg Med. 2024 Apr 29;32:38. doi: 10.1186/s13049-024-01209-x (PMC11059688; doi:10.1186/s13049-024-01209-x)
Supplement: Supplementary file 1 — Supplementary Material 1. [file 13049_2024_1209_MOESM1_ESM.docx]

## Supplement 1

## Articles from literature review

Atack, L., & Maher, J. (2010). Emergency medical and health providers’ perceptions of key issues in prehospital patient safety. *Prehospital Emergency Care*, *14*(1), 95–102. https://doi.org/10.3109/10903120903349887

Bigham, B. L., Buick, J. E., Brooks, S. C., Morrison, M., Shojania, K. G., & Morrison, L. J. (2012). Patient safety in emergency medical services: A systematic review of the literature. *Prehospital Emergency Care*, *16*(1), 20–35. https://doi.org/10.3109/10903127.2011.621045

Bigham, B. L., Bull, E., Morrison, M., Burgess, R., Maher, J., Brooks, S. C., & orrison, L. J. (2011). Patient safety in emergency medical services: Executive summary and recommendations from the niagara summit. *Canadian Journal of Emergency Medicine*, *13*(1), 13–18. https://doi.org/10.2310/8000.2011.100232

Boyle, M. J. (2009). Comparison Overview of Prehospital Errors Involving Road Traffic Fatalities in Victoria, Australia. *Prehospital and Disaster Medicine*, *24*(3), 254–261. https://doi.org/10.1017/S1049023X00006890

Colldén Benneck, J., & Bremer, A. (2019). Registered nurses’ experiences of near misses in ambulance care – A critical incident technique study. *International Emergency Nursing*, *47*, 100776. https://doi.org/10.1016/j.ienj.2019.05.002

Cushman, J. T., Fairbanks, R. J., O’Gara, K. G., Crittenden, C. N., Pennington, E. C., Wilson, M. A., Chin, N. P., & Shah, M. N. (2010). Ambulance Personnel Perceptions of Near Misses and Adverse Events in Pediatric Patients. *Prehospital Emergency Care*, *14*(4), 477–484. https://doi.org/10.3109/10903127.2010.497901

Fairbanks, R. J., Crittenden, C. N., O’Gara, K. G., Wilson, M. A., Pennington, E. C., Chin, N. P., & Shah, M. N. (2008). Emergency Medical Services Provider Perceptions of the Nature of Adverse Events and Near-misses in Out-of-hospital Care: An Ethnographic View. *Academic Emergency Medicine*, *15*(7), 633–640. https://doi.org/10.1111/j.1553-2712.2008.00147.x

Gallagher, J. M., & Kupas, D. F. (2012). Experience with an anonymous web-based state EMS safety incident reporting system. *Prehospital Emergency Care*, *16*(1), 36–42. https://doi.org/10.3109/10903127.2011.626105

Goodloe, J. M., Crowder, C. J., Arthur, A. O., & Thomas, S. H. (2012). EMS Stretcher “Misadventures” in a Large, Urban EMS System: A Descriptive Analysis of Contributing Factors and Resultant Injuries. *Emergency Medicine International*, *2012*, 1–4. https://doi.org/10.1155/2012/745706

Hagiwara, M. A., Magnusson, C., Herlitz, J., Seffel, E., Axelsson, C., Munters, M., Strömsöe, A., & Nilsson, L. (2019). Adverse events in prehospital emergency care: a trigger tool study. *BMC Emergency Medicine*, *19*(1), 14. https://doi.org/10.1186/s12873-019-0228-3

Hohenstein, C., Hempel, D., Schultheis, K., Lotter, O., & Fleischmann, T. (2014). Critical incident reporting in emergency medicine: results of the prehospital reports. *Emergency Medicine Journal*, *31*(5), 415–418. https://doi.org/10.1136/emermed-2012-201871

Howard, I., Howland, I., Castle, N., Al Shaikh, L., & Owen, R. (2022). Retrospective identification of medication related adverse events in the emergency medical services through the analysis of a patient safety register. *Scientific Reports*, *12*(1). https://doi.org/10.1038/s41598-022-06290-9

Howard, I. L., Bowen, J. M., Al Shaikh, L. A. H., Mate, K. S., Owen, R. C., & Williams, D. M. (2017). Development of a trigger tool to identify adverse events and harm in Emergency Medical Services. *Emergency Medicine Journal*, *34*(6), 391–397. https://doi.org/10.1136/emermed-2016-205746

Howard, I., Pillay, B., Castle, N., Al Shaikh, L., Owen, R., & Williams, D. (2018). Application of the emergency medical services trigger tool to measure adverse events in prehospital emergency care: a time series analysis. *BMC Emergency Medicine*, *18*(1), 47. https://doi.org/10.1186/s12873-018-0195-0

Montero García, A., Patricia, O., Guerrero, J., Chaves, E. C., González Aranda, L., García, S., Miguel, J., & Asencio, M. (2020). *Psychometric Design and Validation of an Adverse Event Vulnerability Scale in Prehospital Emergency Care*. http://links.lww.com/JPS/A261

Patterson, P. D., Lave, J. R., Martin-Gill, C., Weaver, M. D., Wadas, R. J., Arnold, R. M., Roth, R. N., Mosesso, V. N., Guyette, F. X., Rittenberger, J. C., & Yealy, D. M. (2014). Measuring Adverse Events in Helicopter Emergency Medical Services: Establishing Content Validity. *Prehospital Emergency Care*, *18*(1), 35–45. https://doi.org/10.3109/10903127.2013.818179

Patterson, P. D., Weaver, M. D., Abebe, K., Martin-Gill, C., Roth, R. N., Suyama, J., Guyette, F. X., Rittenberger, J. C., Krackhardt, D., Arnold, R., Yealy, D. M., & Lave, J. (2012). Identification of adverse events in ground transport emergency medical services. *American Journal of Medical Quality*, *27*(2), 139–146. https://doi.org/10.1177/1062860611415515

Price, R., Bendall, J. C., Patterson, J. A., & Middleton, P. M. (2013). What causes adverse events in prehospital care? A human-factors approach. *Emergency Medicine Journal*, *30*(7), 583–588. https://doi.org/10.1136/emermed-2011-200971

Shaw, R., Drever, F., Hughes, H., Osborn, S., & Williams, S. (2005). Adverse events and near miss reporting in the NHS. *Quality and Safety in Health Care*, *14*(4), 279–283. https://doi.org/10.1136/qshc.2004.010553

Spangler, D., Edmark, L., Winblad, U., Colldén-Benneck, J., Borg, H., & Blomberg, H. (2020). Using trigger tools to identify triage errors by ambulance dispatch nurses in Sweden: an observational study. *BMJ Open*, *10*(3), e035004. https://doi.org/10.1136/bmjopen-2019-035004

Stella, J., Bartley, B., & Jennings, P. (2010). Introduction of a Prehospital Critical Incident Monitoring System—Final Results. *Prehospital and Disaster Medicine*, *25*(6), 515–520. https://doi.org/10.1017/S1049023X00008694

Wang, H. E., Fairbanks, R. J., Shah, M. N., Abo, B. N., & Yealy, D. M. (2008). Tort Claims and Adverse Events in Emergency Medical Services. *Annals of Emergency Medicine*, *52*(3), 256–262. https://doi.org/10.1016/j.annemergmed.2008.02.011

Wang, H. E., Weaver, M. D., Abo, B. N., Kaliappan, R., Fairbanks, R. J., & Wang, E. (2009). Ambulance stretcher adverse events. *Qual Saf Health Care*, *18*, 213–216. https://doi.org/10.1136/qshc.2007.024562

Wolff, A. M. (2002). Detecting and reducing adverse events in an Australian rural base hospital emergency department using medical record screening and review. *Emergency Medicine Journal*, *19*(1), 35–40. https://doi.org/10.1136/emj.19.1.35

Yardley, I. E., & Donaldson, L. J. (2016). Deaths following prehospital safety incidents: An analysis of a national database. *Emergency Medicine Journal*, *33*(10), 716–721. <https://doi.org/10.1136/emermed-2015-204724>
