# Supplementary material for: Development of a trigger tool to identify harmful incidents, no harm incidents, and near misses in prehospital emergency care
Source: Scand J Trauma Resusc Emerg Med. 2024 Apr 29;32:38. doi: 10.1186/s13049-024-01209-x (PMC11059688; doi:10.1186/s13049-024-01209-x)
Supplement: Supplementary file 2 — Supplementary Material 2. [file 13049_2024_1209_MOESM2_ESM.docx]

# Supplement 2

### Progression of triggers in each session of structured discussions

| **Baseline triggers** | **Session one** | **Session two** | **Session three and the final ATT** |
| --- | --- | --- | --- |
| A1 Incomplete documentation | A1 Incomplete documentation | A1 Incomplete documentation | A1 Incomplete documentation |
| A2 Patient remains at home | A2 Response Time >20 minutes for priority 1 | A2 Response Time >20 minutes for priority 1 | A2 Response Time >20 minutes for priority 1 (lights and sirens) |
| A3 Lack of continued planning for the patient who remains at home (e.g. when renewed care contact should be made) | A3 Time on site >10 min in life-threatening conditions | A3 Time on site >10 min in life-threatening conditions | A3 Time on site >10 min in life-threatening conditions |
| A4 Patient contacts ED within 48 h after assessment by EMS | A4 Weather and environment affect patient care | A4 Weather and environment affect patient care | A4 Breakdown or faulty/missing equipment |
| A5 Accident of ambulance during ongoing mission | A5 Breakdown or faulty/missing equipment | A5 Breakdown or faulty/missing equipment | A5 Shortage of ambulance resources |
| A6 Faulty technical equipment | A6 Shortage of ambulance resources | A6 Shortage of ambulance resources | A6 Other |
| A7 Patient fall | A7 Other | A7 Other | B1 Deviations from treatment guidelines |
| A8 Documentation of mistakes | B1 Deviations from treatment guidelines | B1 Deviations from treatment guidelines | B1A Assessment/Interventions according to SX-ABCDE |
| A9 Other | B1A Assessment/Interventions according to SX-ABCDE | B1A Assessment/Interventions according to SX-ABCDE | B1B Assessment/Interventions for specific conditions |
| B1 Deterioration of Patients Condition during Transport | B1B Assessment/Actions in specific conditions | B1B Assessment/Interventions for specific conditions | B1C Absence of measured vital signs |
| B2 Deviation from treatment guidelines | B1C Absence of measured vital signs | B1C Absence of measured vital signs | B1D Absence of relevant clinical examination |
| B2A Assessment/Interventions according to SX-ABCDE | B1D Absence of relevant clinical examination | B1D Absence of relevant clinical examination | B2 Physical injury during patient transport |
| B2B Absence of measured vital signs | B2 Physical injury during patient transport | B2 Physical injury during patient transport | B3 Deterioration of Patients Condition during Transport |
| B2C Absence of relevant clinical examination | B3 Deterioration of Patients Condition during Transport | B3 Deterioration of Patients Condition during Transport | B4 Telephone interpreter has not been used in case of language deficiency |
| B2D Lack of stabilization/spinal movement limitation in fractures and trauma | B4 Assessment and triage are affected due to language deficiencies | B4 Assessment and triage are affected due to language deficiencies | B5 Inconsistency between the EMS clinicians and emergency physicians assessment and triage |
| B2E Other deviations | B5 The ambulance nurse’s assessment and triage are not compatible with the emergency physician’s assessment | B5 The ambulance nurse’s assessment and triage are not compatible with the emergency physician’s assessment | B6 The patient is non conveyed after EMS assessment |
| B5 Ambulance nurse’s assessment and triage not consistent with emergency physician’s assessment | B6 The patient is non conveyed after EMS assessment | B6 Patient care is terminated after the ambulance nurse’s assessment | B7 Alternative mode of transport to definitive care |
| B6 Assessment and triage through family interpreter | B7 Alternative mode of transport to definitive care | B7 Alternative mode of transport to definitive care | B8 Ambulance destination deviates from local guidelines |
| B7 Invasive/surgical interventions and compressions with abnormal results | B8 Ambulance destination deviates from local guidelines | B8 Ambulance destination deviates from local guidelines | L1 Unfavourable/Inappropriate drug treatment |
| B8 Patient’s condition exceeds the competence of the ambulance nurse | L1 Unfavourable/Inappropriate drug treatment | L1 Unfavourable/Inappropriate drug treatment |  |
| T1 Forwarding time longer than acceptable level | L2 Mix-up of drugs | L2 Mix-up of drugs |  |
| T2 Time on site exceeds acceptable level in life-threatening conditions | L3 Shortage of medicines due to absence/expiry date | L3 Shortage of medicines due to absence/expiry date |  |
| T3 Hospital selection negatively affects the patient’s condition |  |  |  |
| T4 Secondary transport to higher level of care |  |  |  |
| T5 Weather and environment affect patient care |  |  |  |
| T6 Shortage of ambulance resources |  |  |  |
| T7 Collision with ambulance |  |  |  |
| T8 The patient is injured when moving |  |  |  |
| T9 Wrong address from alarm centre |  |  |  |
| T10 The patient is transported to the emergency room with a relative/sick trip |  |  |  |
| L1 Medicine shortage/expiry date |  |  |  |
| L2 Anaphylactic reaction |  |  |  |
| L3 Unfavourable effect of drug therapy |  |  |  |
| L4 Mix-up of drugs |  |  |  |
