# Supplementary material for: Development of a trigger tool to identify harmful incidents, no harm incidents, and near misses in prehospital emergency care
Source: Scand J Trauma Resusc Emerg Med. 2024 Apr 29;32:38. doi: 10.1186/s13049-024-01209-x (PMC11059688; doi:10.1186/s13049-024-01209-x)
Supplement: Supplementary file 3 — Supplementary Material 3. [file 13049_2024_1209_MOESM3_ESM.pdf]

Retrospective record review with a trigger tool

TRIGGERS AND DEFINITIONS EMS EDITION

Disclaimer: This edition has been translated from Swedish by the author and not in any professional manner.

Content

Definitions 2
General Triggers 3
A1 Incomplete documentation 3
A2 Response Time >20 minutes for priority 1 (lights and sirens) 4
A3 Time on site >10 minutes in case of life-threatening conditions 4
A4 Breakdown or faulty/missing equipment 5
A5 Shortage of ambulance resources 5
A6 Other 6
Assessment/Intervention Triggers 6
B1 Deviations from treatment guidelines 6
B1A Assessment/Interventions according to SX-ABCDE 7
B1B Assessment/Interventions for specific conditions 9
B1C Absence of measured vital signs 9
B1D Absence of relevant clinical examination 10
B2 Physical harm during patient transport 11
B3 Deterioration of patients condition during transport 12
B4 Telephone interpreter has not been used in case of language deficiency 13
B5 Inconsistency between the EMS clinicians and emergency physicians assessment and triage 14
B6 The patient is non conveyed after EMS assessment 15
B7 Alternative mode of transport to definitive care 18
B8 Ambulance destination deviates from local guidelines 19
Trigger related to drug administration 20
L1 Unfavorable/Inappropriate drug treatment 20

Definitions

Positive trigger

Indication that an incident has occurred and needs evaluation by the reviewer.

Incident

An event that is considered deviant and categorizes into near misses, no harmful incident and harmful incident according to the WHO Conceptual Framework for the International Classification for Patient Safety.

Near miss (NM)

An NM incident (e.g. lack of documentation) neither affects nor harms the patient but poses the risk of an error and is categorized as (A) or (B) according to the NCC MERP.

No harmful incident (NHI)

An NHI reaches the patient but does not cause harm (e.g. omission of electrocardiogram (ECG) in patient with chest discomfort not diagnosed with acute coronary syndrome) affects the patient but does not cause harm. It is categorized as (C) 'An incident that affected the patient but did not cause any harm' or (D) 'An incident that affected the patient and demanded observation or treatment to assure that no harm occurred'.

Harmful incident (HI, adverse event)

A HI is an incident that results in harm the patient (adverse event; e.g. omission of ECG in patient with chest discomfort and later diagnosed with ST-elevated myocardial infarction, thus delaying time to causal treatment) harms the patient. It is categorized as (E) 'Contributed to or resulted in temporary harm and required intervention'; (F) 'Contributed to or resulted in temporary harm requiring outpatient care, readmission, or prolonged hospital care'; (G) 'Contributed to or caused permanent patient harm'; (H) 'An event that required lifesaving intervention within 60 min'; or (I) 'Contributed to the patient's death'.

The process of RRR with a trigger tool

The primary reviewer reviews the record for positive triggers. If positive triggers are found, an assessment is made by the primary reviewer as whether the positive trigger contributed to an incident or not and if it affected the patient or not. Incidents that did not affect the patient or entail any risk of harm are classified according to the steps AB and C (Incidents Table 1). An incident with a risk of harm to the patient is subject to a secondary review by a physician, and the incident is not classified in Table 1 by the primary reviewer but is left to the secondary reviewer. The secondary reviewer assesses incidents with a risk of harm and decides whether the patient has been harmed or not. If the patient has not been harmed, the scale steps no incident, AB, C and D are used (Table 1). If the patient has been harmed, the type of harm (Table 2), the severity according to scale steps E to I (Table 3) and whether the harm was preventable or not (Table 4) is documented.

General Triggers

A1 Incomplete documentation

**Definition**	**Incomplete documentation**
**Considerations**	Ambulance personnel are often the first healthcare providers to encounter the patient. The assessment by the paramedic can be crucial for the patients ongoing contact with healthcare.<br><br>The trigger is considered positive if any of the following are missing:<br><br>• Information about the patients identity<br><br>• Essential details about the background leading to contact with EMS<br><br>• Information about the diagnosed condition and the reason for more significant measures<br><br>• Essential details about measures taken and planned actions<br><br>• Information about the communication provided to the patient and the decisions made regarding the choice of treatment options and the possibility of a renewed medical assessment
**Incident**	Positive trigger is always considered an incident
**Harm**	Incomplete documentation does not by itself have to cause harmful incident but is an important measure of quality.
**Preventabillity**	Incomplete documentation is considered a incident that is always avoidable.

A2 Response Time >20 minutes for priority 1 (lights and sirens)

**Definition**	**Prio 1 >20 minutes or other time agreed locally, for example rural areas**
**Considerations**	The trigger is considered positive if:<br>• The response time to the patient exceeds 20 minutes, calculated from ambulance alert to arrival at the address, regardless of the cause
**Incident**	A positive trigger is always considered an incident.
**Harmful incident**	Time-critical condition that are delayed.
**Preventabillity**	Incident may be considered preventable if:<br>• The nearest available resource is already beyond >20 minutes at the time of dispatch.

A3 Time on site >10 minutes in case of life-threatening conditions

**Definition**	**Time on site >10 minutes in case of life-threatening conditions**
**Considerations**	In cases of life-threatening conditions, the paramedic has limited resources for definitive treatment. Initiating transport is crucial for conditions or injuries that can only be addressed at the hospital.<br><br>The trigger is considered positive if:<br>• Time on site >10 minutes from arriving at the patient to loading for a priority 1 transport to definitive care.
**Incident**	A positive trigger is always considered an incident.
**Harm incidents**	Delay to definitive treatment.
**Preventabillity**	Incidents are considered avoidable if:<br>• The conditions have been in place to be able to transport the patient within 10 minutes, but there has been a delay for no obvious reasons.

A4 Breakdown or faulty/missing equipment

**Definition**	**Breakdown of ambulance or faulty/missing technical/medical equipment which affects the patients assessment/treatment**
**Considerations**	The trigger is considered positive if:<br>• Breakdown of the ambulance that requires an additional ambulance to manage the patient.<br>• Faulty/missing technical equipment or medicines, such as ECG, not readable or cannot be sent to a higher level of care, lack of map support<br>• Collision.
**Incident**	A positive trigger is always considered an incident.
**Harm incidents**	It may not necessarily imply harm to the patient, but substandard assessment or treatment due to incorrect or missing equipment is a quality deficiency, and other types of harm may be result.
**Preventabillity**	Incidents shall be considered preventable if:<br>• Ambulance vehicle servicing is not done.<br>• Incorrect or missing equipment that could have been detected during ambulance functional checks, such as an empty oxygen cylinder, forgotten emergency bag, missing/expired medications.

A5 Shortage of ambulance resources

**Definition**	Patient(s) demand of care exceeds available resources.
**Considerations**	The trigger is considered positive if:<br>• The EMS clinicians requests additional resources but are not acquired due to a shortage of resources.<br>• A lower level of ambition than documented treatment guidelines is applied due to a shortage of resources.
**Incident**	A positive trigger is always considered an incident.
**Harm incidents**	Patient(s) do not receive treatment according to guidelines due to lack of resources.
**Preventabillity**	Incidents/harm are always considered preventable.

A6 Other

**Definition**	**Any incident that is not covered by another trigger**
**Considerations**	Incidents that are not identified by any other trigger are noted.<br><br>Describe the incident.

Assessment/Intervention Triggers

B1 Deviations from treatment guidelines

**Definition**	**Deviations from treatment guidelines**
**Considerations**	The EMS clinician uses an examination methodology according to SX-ABCDE and different clinical examination in relation to chief complaint, as well as various tools to assess and treat the patient presenting with various conditions. The purpose is to identify serious conditions where supportive treatment can be provided.<br><br>The trigger is considered positive if:<br><br>• The reviewer has reason to believe that deviations from treatment guidelines have been made. Further classification is made according to B2A, B2B or A6.
**Harm incidents**	Time-critical conditions such as stroke, myocardial infarction, sepsis, spinal cord injuries with an incorrect level of care that delays treatment with a risk of harm.
**Preventabillity**	Incidents shall be considered preventable if:<br><br>There is no motivation why deviations from treatment guidelines have been made.

B1A Assessment/Interventions according to SX-ABCDE

**Definition**	Deviation from treatment guidelines involving assessment and interventions according to SX-ABCDE.
**Considerations**	EMS clinicians utilizes an examination methodology according to SX-ABCDE, aiming to identify and address immediate life threats.<br><br>The trigger is considered positive if:<br><br>• Scene safety (S). Example: Lack of consideration for the scene where the patient is being cared for.<br>• Catastrophic (X, arterial). Example: Failure to stop bleeding with pressure dressing/ tourniquet (TQ). Wrong indication/application of TQ.<br>• Airway obstruction (A). Example: Failure to establish a clear airway using simple maneuvers or basic/advanced airway tools, including surgical airway or the reviewer perceives that airway management has not been done correctly.<br>• Ventilation issues (B). Example: Oxygen is not provided to a hypoxic patient, apnea/hypoventilation/hypoxia despite oxygen treatment not addressed with assisted breathing/CPAP or needle decompression, thoracostomy/thoracotomy performed for incorrect indications or the reviewer perceives that the management has not been done correctly.<br>• Circulatory issues (C). Example: Shock of various origins not addressed/treatment not initiated, incorrect placement of mechanical compressions, intra-arterial or subcutaneous placement of IV catheter, intraosseous needle with incorrect placement. The reviewer perceives that the management has not been done correctly.<br>• Neurological issues (D). Example: Decreased consciousness with a reversible cause for example hypoxia, hypoglycemia, intoxication not addressed or the reviewer perceives that the management has not been done correctly.<br>• Exposure issues (E). Example: Severely hypothermic patient where warming has not started or the reviewer perceives that the management has not been done correctly.

	Note that SX-ABCDE does not necessarily need to be written out in the record but can be assessed by the reviewer. For example, if history and vital parameters are present, an assessment can be made that the algorithm is under control.
**Incident**	A positive trigger is always considered an incident.
**Harm incidents**	Airway injuries, pneumothorax/hemothorax, damage to internal organs, thrombophlebitis.
**Preventabillity**	Incidents shall be considered preventable if:<br>• Care in the hazardous zone without action or improper action.<br>• Catastrophic bleeding without action or improper action.<br>• Respiratory problems without action or improper action.<br>• Ventilation problems without action or improper action.<br>• Circulatory problems without action or improper action.<br>• Neurological problems (loss of consciousness) without action or improper action.<br>• Exposure (protection against the hazard at the scene) without action or improper action.<br>• Improper actions due to lack of competence of the EMS clinician, have additional resources been requested or higher medical competence consulted?

B1B Assessment/Interventions for specific conditions

Definition	**Deviations from treatment guidelines for specific conditions that do not fall under interventions according to SX-ABCDE**
**Considerations**	• The trigger is considered positive if, for example:<br>• Absence of spinal movement restriction/stabilization when indicated.<br>• Aspirin not administered to a patient with a high suspicion of acute coronary syndrome.<br>• Glucocorticoid not given in suspected Addison crisis.<br>• Failure to provide pain relief when VAS>4.<br>• Or if the reviewer identifies other deviations.
**Incident**	A positive trigger is always considered an incident.
**Preventabillity**	Incidents shall be considered preventable:<br>• Improper interpretation of ECG.<br>• Absence of spinal movement restriction/stabilization when indicated or applied improperly. For example, the SRB significantly delays the time spent on scene in the event of life-threatening injuries.<br>• Improper actions due to lack of competence of the EMS clinician, have additional resources been requested or higher medical competence consulted?

B1C Absence of measured vital signs

Definition	**Respiratory rate, saturation, blood pressure, pulse, level of consciousness and temperature are missing from documentation.**
**Considerations**	EMS largely relies on assessing and treating patients based on their vital signs.<br><br>Triggers are considered positive if any part of the vital signs is missing.<br><br>P-glucose is included here if the chief complaint indicates the control of p-glucose, such as decreased consciousness or seizures.
**Incident**	A positive trigger is always considered an incident.
**Harm incidents**	Absence of vital signs where the next healthcare provider detects abnormal vital signs that require treatment.

**Preventabillity**	Incidents shall be considered preventable:<br>• The measure of vital signs has not been performed and a relevant motivation as to why is missing.

B1D Absence of relevant clinical examination

**Definition**	**Absence of clinical examinations relevant to the patients chief complaint**
**Considerations**	EMS clinicians relies on clinical examinations of the patient for their assessment and treatment.<br><br>Trigger are considered positive if:<br><br>Relevant clinical examinations are missing depending on the chief complaint. Note that a nonspecific chief complaint, multiple examinations may be considered relevant.
**Incident**	A positive trigger is always considered an incident.
**Harm incidents**	Conditions that could be detected by clinical examination such as: stroke, myocardial infarction, arrhythmias, surgical diseases of the abdomen.
**Preventabillity**	Incidents shall be considered preventable if relevant clinical examinations have not been performed, such as:<br>• ECG not performed for chest pain, dyspnea, syncope or high abdominal pain.<br>• Pulmonary auscultation in dyspnea.<br>• A neurological examination according to an accepted scale is not performed if neurological symptoms are suspected.<br>• Abdominal examination (auscultation, palpation) not performed in abdominal pain.

B2 Physical harm during patient transport

Definition	Physical harm during patient transport
**Considerations**	EMS encounters patients with an elevated risk of falling. The risk of falling can be due to various reasons, such as impairment of sensory function, chronic or acute illness.<br><br>The trigger is considered positive if:<br><br>• Harm occurs during transfer, such as a fall, dropped stretcher, or crush injuries.
**Incident**	A positive trigger is always considered an incident.
**Harm incidents**	Fracture, bleeding, laceration, soft tissue or joint pain, post-concussion symptoms.
**Preventabillity**	Incidents should be considered preventable if:<br><br>• The patient falls in the presence of the EMS clinicians.<br>• The aid of transfer is used incorrectly.

B3 Deterioration of patients condition during transport

**Definition**	Deterioration of the patients vital signs from previously measured values.<br>The trigger is considered positive if <u>at least one</u> of the following is met:<br>**Respiration**<br>• Respiratory arrest<br>• Saturation <90% with oxygen<br>• Respiratory rate <8/min<br>• Respiratory rate >30/min<br>**Cirkulation**<br>• Cardiac arrest<br>• Systolic blood pressure <90 mm Hg<br>• Pulse <40/min<br>• Pulse >140/min<br>**Neurologists**<br>• Glasgow Coma Scale: Fall >2 from baseline<br>• RLS >3<br>• Deterioration according to AVPU<br>• P-glukos <3 mmol/L
**Considerations**	Investigate the events preceding to the deterioration of the patients condition. For example, ingestion of medication, self-mobilization, or a fall. Consider whether there is reason to believe that the deterioration was influenced by the EMS clinicians treatment or lack of treatment.
**Incident**	A positive trigger is always considered an incident. Should be secondary reviewed.
**Harm incidents**	Effects on respiration and circulation regardless of condition. For example, COPD/Asthma, Infection/sepsis, myocardial infarction, heart failure/pulmonary edema, trauma/bleeding.
**Preventabillity**	Incidents/injuries shall be considered avoidable if, among other things:<br>• Monitoring of the patient has not been done according to treatment guidelines or in accordance with the patients condition.<br>• Vital signs have not been adequately recognized which has led to treatment being avoided or not initiated in a reasonable time.

**Metrics and scales associated with this trigger**	Respiration: respiratory rate and saturation.<br>Circulation: systolic blood pressure and pulse.<br>Neurology: Glasgow Coma Scale, RLS, AVPU, P-glucose

B4 Telephone interpreter has not been used in case of language deficiency

**Definition**	**The EMS clinician is unable to communicate with the patient due to a language barrier and uses a relative to gather the necessary information.**
**Considerations**	Patient history is one of the cornerstones in the assessment and continued management of the patient in prehospital care. Telephone interpretation with a relative can both exaggerate and minimize current issues and, therefore, affect the patients management.<br><br>The trigger is considered positive if:<br><br>• There were language deficiencies, and a telephone interpreter was not used. Key factors in the medical history are missing or distorted.
**Incident**	A positive trigger is always considered an incident.
**Harm incidents**	Any harmful incident that can be traced to an inadequate medical history.
**Preventabillity**	Using a relative to the patient as an interpreter should be considered avoidable if:<br>• The situation allows contact with a telephone interpreter.

B5 Inconsistency between the EMS clinicians and emergency physicians assessment and triage

**Definition**	**For time-critical/serious conditions, there is a lack of agreement between the EMS clinicians and the emergency physicians assessment and triage.**
**Considerations**	The purpose of emergency care is to identify serious and time-critical conditions in the patient. EMS have limited capabilities for advanced diagnostics, and patient prioritization (triage) is based on medical history, vital signs, symptoms, and a few clinical examinations.<br><br>The trigger is considered positive if:<br><br>• The patients triage colour deviates from the emergency physician's assessment in hospital, which delays the time to definitive treatment. For example: The patient has been given green/yellow priority but the patient is taken from the emergency department directly to definitive treatment such as PCI, thrombolysis, thrombectomy, emergency surgery or intensive care.<br><br>The purpose is to assess whether under triage has occurred in the EMS.
**Incident**	A positive trigger is always considered an incident.
**Harm incidents**	Serious conditions that are not found in EMS and the patient is given a low priority compared to the emergency physician's final diagnosis.
**Preventabillity**	A low triage related to the patients condition shall be considered preventable if:<br>• The EMS clinician has made a reasonable assessment, but the system for triage did not triage the patient correctly.<br>• The EMS clinician finds/suspects a serious condition in their examination, but the patient is given a green/yellow priority due non acceptance to PCI or intervention regarding stroke.<br>• The EMS clinician has assessed a lower triage than the triage system suggests.

B6 The patient is non conveyed after EMS assessment

**Definition**	**The patient is assigned an ambulance resource, resulting in the termination of care based on the EMS clinicians assessment**
**Considerations**	Patients who remain at home pose a risk of unmonitored deterioration or the possibility that a serious illness has not been identified in the paramedic's assessment.<br><br>The trigger is considered positive if:<br><br>• The patient is non conveyed.<br><br>Note what was behind the decision, was the contact terminated by the ambulance nurse or the patient, was the patient competent to make a decision, i.e. could understand the consequences of the decision, was there a further planning for follow-up of, for example, primary care, in case of which symptoms should the patient contact the ambulance service again. Also note if the patient sought emergency care within 72 hours for the same symptoms and action at the time of care.<br><br>Note what led to the decision, whether the contact was terminated by the EMS or the patient, whether the patient was competent to make a decision (understand the consequences of the decision), if there was a further plan for follow-up, such as with primary care, and at what symptoms the patient should contact EMS again. Also, note if the patient contacted emergency care (EMS, emergency department) within 72 hours for the same symptoms again and if any intervention took place.
**Incident**	A positive trigger is an incident if the patient contacts emergency care again within 72 hours for the same symptoms. Severity depends on the intervention done.
**Harmful incident**	Any condition where time to treatment is of importance.
**Preventabillity**	Incidents is considered preventable if:<br><br>• It appears that the patient has been motivated by the EMS to remain at home or contact a level of care where necessary interventions are not available. For instance, Chief complaint chest pain referred are to primary care.<br>• It appears that the patient needed emergency care but was non convenyed.

B7 Alternative mode of transport to definitive care

**Definition**	**The patient is transported to the emergency department by alternative mode of transport instead of ambulance after EMS assessment**
**Considerations**	The trigger is considered positive if:<br><br>• The patient is transported to the ED by alternative mode of transport. Does not necessarily involve an incident but is a risk of unattended deterioration and lack of treatment during transport.
**Incident**	A positive trigger is an incident if the patients condition deteriorates during the alternative mode of transport.
**Harmful incident**	Injuries that occur due to lack of treatment and extended processing time.
**Preventabillity**	Incidents that occur in connection with an alternative mode of transport are considered preventable if:<br><br>• Chief complaint and/or condition warranted transport by ambulance, but alternative mode of transport is still chosen.

B8 Ambulance destination deviates from local guidelines

**Definition**	**The choice of hospital extends the patients time to definitive treatment. Definitive treatment refers to procedures such as PCI for a heart attack, thrombolysis for a stroke, neurosurgery for severe head injuries**
**Considerations**	EMS and the availability of specialist competencies vary depending on the region and population density. In urban areas, specialist competencies are available but are often concentrated in specific hospitals. It is therefore crucial for the patient to be transported directly to definitive treatment. There are, of course, situations where the nearest hospital should be chosen, such as in the case of an obstructed airway, but the general rule is that hospitals capable of providing definitive treatment should be prioritized. This may not be possible in rural areas.<br><br>The trigger is considered positive if:<br><br>• Despite an apparent need for specialist competence, where local guidelines prescribe transport to a specific hospital, the patient is transported to a hospital lacking this competence without a motivation for the decision.<br>• Secondary transport to a higher level of care.
**Incident**	A positive trigger always results in an incident. Severity depends on how the patient has been affected.
**Harmful incident**	Extended time to definitive treatment such as heart attack, stroke, head injuries, high-energy trauma with multiple injuries.
**Preventabillity**	Incident is considered preventable if:<br><br>• The patient is transported to a hospital without specialist competence despite an apparent need, and there is no motivation behind the decision.

Trigger related to drug administration

L1 Unfavorable/Inappropriate drug treatment

**Definition**	**Signs of unfavorable effects of drug treatment, such as hypersensitivity/anaphylaxis, antidote administration, inappropriate drug/dose, or incorrect administration method**
**Considerations**	Drug treatment can have negative effects on organs and vital functions. This may require the treatment to be unexpectedly discontinued or the need for treatment to counteract the negative effects. Among the drugs used to reverse the negative effects are specific antidotes such as naloxone (opioid overdose) and flumazenil (benzodiazepine overdose).<br><br>The trigger is considered positive if:<br>• Interventions regarding ABCD following drug administration<br>• The use of antidote following drug administration<br>• Allergy/anaphylaxis following drug administration<br>• The patient is administered the wrong drug<br>• If the reviewer experiences inappropriate drug treatment/dosage
**Incident**	Positive trigger always result in an incident.
**Harmful incident**	Hypersensitivity reaction, confusion, decreased consciousness, unconsciousness, respiratory distress, respiratory arrest, circulatory failure, and death.
**Preventabillity**	Incidents shall be considered preventable if:<br><br>• Medication is given despite contraindication or known hypersensitivity.<br>• The risk of unfavorable effects has not been considered regarding interacting drugs.<br>• Treatment with opioids or benzodiazepines has caused symptoms requiring antidote administration (naloxone, flumazenil).<br>• Transfusion reaction caused by mistakes during preparation for or in connection with the transfusion.

Step-by-step, user guide for retrospective record review with a trigger tool

1) **Identify records for review:**
   - Select records for review, either through random sampling or targeted selection.
2) **Use a review form for each record:**
   - Utilize a review form for each record, assigning a unique sequential number from a designated series that you create.
3) **Document patient ID and unique number:**
   - Document the patient's ID along with the specific sequential number on a list maintained by the review team under strict journal confidentiality. This list facilitates the identification of an individual care instance if further analysis is required in future development work.
4) **Search for positive triggers in selected records:**
   - Examine the records for positive triggers. Refer to the definitions in the document on triggers and definitions. Mark positive trigger in the review form with a "+" sign, and note where in the record documentation the trigger was found, along with the reason for the positive trigger.
5) **Primary reviewers notation of triggers and incidents:**
   - The primary reviewer reviews the record for positive triggers. If positive triggers are found, an assessment is made by the primary reviewer as whether the positive trigger contributed to an incident or not and if it affected the patient or not. Incidents that did not affect the patient or entail any risk of harm are classified according to the steps AB and C (Incidents Table 1). An incident with a risk of harm to the patient is subject to a secondary review by a physician, and the incident is not classified in Table 1 by the primary reviewer but is left to the secondary reviewer.
6) **Secondary reviewers assessment of incidents with risk of harm:**
   - The secondary reviewer assesses incidents with a risk of harm and determines whether the patient was harmed or not. If the patient was not harmed, the no incident, AB, C, and D scales (Table 1) are used. If the patient was harmed, the type of harm is assessed (Table 2), severity (Table 3) according to scales E to I, and whether the harmful incident was preventable or not (Table 4).
7) **Documentation and summary of the record review:**
   - The RRR is documented in the review form and summarized, forming the basis for analysis and planning risk-reducing measures to enhance patient safety.

Assignment to work with retrospective record review with a trigger tool

____

EMS organisation

____

Name *personal identity number*

As of date, I have ______ been commissioned on behalf of the ambulance service to carry out retrospective record review with a trigger tool as part of the work for increased patient safety. This work includes taking part of information in the various medical record and documentation systems that the ambulance service has access to. The methodology for retrospective record review means that medical records from the ambulance service are subject to review and, in cases where the care occasion is also directly related to care at other clinics. The time required by the above person to carry out his/her assignment is calculated to ______ hours per month. The assignment is valid until the end of the year. ______

____

Head of Operations (signature and clarification) *(date)*

____

EMS chief physician/equivalent (signature and clarification)

(date)

The assignment is drawn up in three identically signed copies, where the contractor's delegee, Head of Operations and the EMS Chief Physician/equivalent each keep one copy.

Review form, retrospective record review with trigger tool for EMS

Serial number: __________ Case number ____________

Time for EMS mission: _________

<table>
<tr><th></th><th>General Triggers</th><th>#</th><th colspan="5">Reason for positive trigger/ where in the record was the trigger found?</th></tr>
<tr><td>A1</td><td>Incomplete documentation</td><td></td><td colspan="5"></td></tr>
<tr><td>A2</td><td>Response time >20 minutes for priority 1</td><td></td><td colspan="5"></td></tr>
<tr><td>A3</td><td>Time on site >10 minutes in case of life-threatening condition</td><td></td><td colspan="5"></td></tr>
<tr><td>A4</td><td>Breakdown or faulty/missing equipment</td><td></td><td colspan="5"></td></tr>
<tr><td>A5</td><td>Shortage of ambulance resources</td><td></td><td colspan="5"></td></tr>
<tr><td>A6</td><td>Other</td><td></td><td colspan="5"></td></tr>
<tr><th></th><th>Assessment/Intervention Triggers</th><th></th><th colspan="5"></th></tr>
<tr><td>B1</td><td>Deviations from treatment guidelines</td><td></td><td colspan="5"></td></tr>
<tr><td>B1A</td><td>Assessment/interventions according to SX-ABCDE</td><td></td><td colspan="5"></td></tr>
<tr><td>B1B</td><td>Assessment/interventions for specific conditions</td><td></td><td colspan="5"></td></tr>
<tr><td>B1C</td><td>Absence of vital signs</td><td></td><td colspan="5"></td></tr>
<tr><td>B1D</td><td>Absence of relevant clinical examination</td><td></td><td colspan="5"></td></tr>
<tr><td>B2</td><td>Physical harm during patient transport</td><td></td><td colspan="5"></td></tr>
<tr><td>B3</td><td>Deterioration of patients condition during transport</td><td></td><td colspan="5"></td></tr>
<tr><td>B4</td><td>Telephone interpreter has not been used in case of language deficiency</td><td></td><td colspan="5"></td></tr>
<tr><td>B5</td><td>Inconsistency between the EMS clinicians and emergency physicians assessment and triage</td><td></td><td colspan="5"></td></tr>
<tr><td>B6</td><td>The patient is non conveyed after EMS assessment</td><td></td><td>Cause</td><td>72H</td><td>Intervention</td><td>Hospitalized</td><td>Discharged</td></tr>
<tr><td>B7</td><td>Alternative mode of transport to definitive care</td><td></td><td colspan="5"></td></tr>
<tr><td>B8</td><td>EMS destination deviates from local guidelines</td><td></td><td colspan="5"></td></tr>
</table>

Continued. Review form, retrospective record review with trigger tool for EMS

Serial number: ____________ Case number: ______________

Time for EMS mission: __________

	Trigger related to drug administration		
L1	Unfavorable/inappropriate drug treatment		

Number of incidents<br>Primary reviewer	________________	Classification of incidents<br>Primary reviewer<br>(Table 1)	___________	Incident with a risk of harm.<br>Yes/No<br>Secondary review by physician	_____________
Harmful incident?<br>Yes/No	________________	Classification of incidents<br>Secondary reviewer<br>(Table 1)<br>Use if harmful incident No<br>Stop if no harm identified	___________	Type of harm<br>(Table 2)<br>Use if harmful incident Yes<br>Continue with categorization of severity	_____________
Categorisation of severity<br>(Table 3)	________________	Assessment of Preventabillity<br>(Table 4)	___________	Number of harmful incidents	_____________

Classification of incidents, types of harm, severity, and preventability

Table 1. Classification of incidents	
No incident	No incident in record
Category AB	An incident that may cause error (risk) but did not affect the patient. Example; Inadequate documentation, lack of examinations, vital signs.
Category C	An incident that affected the patient but did not cause any harm. Example; Active treatments/interventions with undesirable results. No harm identified
Category D	An incident that affected the patient and demanded observation or treatment to assure that no harm occurred. Example; Active treatments/interventions with undesirable results where active interventions are required to ensure no harm.

	Table 2. Types of harm
1	Allergic reaction
2	Bleeding, not in connection with surgery or other invasive procedure
3	Bleeding, in connection with surgery or other invasive procedure
4	Harm induced by fall
5	Skin damage or superficial vascular damage
6	Infections incl. thrombophlebitis
7	Harm to organ
8	Failure of vital signs including cardiac arrest
9	Anesthesia-related harm
10	Drug-related harm (non-allergic reaction)
11	Harm caused by medical technology
12	Postpartum/obstetric harm
13	Neurological harm
14	Harm to thorax
15	Harm to extremity
16	Delayed care
17	Other damages, specify

Table 3. Classification of harm severity	
Category E	Contributed to or resulted in temporary harm that required intervention.
Category F	Contributed to or resulted in temporary harm that required outpatient care, readmission, or prolonged hospital care.
Category G	Contributed to or caused permanent harm.
Category H	An event that required lifesaving intervention within 60 min.
Category I	Contributed to the patients death.

Table 4. Assessment of preventabillity	
1	Harm *was not* preventable
2	Harm *was unlikely to be* preventable
3	Harm was likely preventable
4	Harm was preventable
